# UPLC/Q-TOF MS-based femoral muscle metabolomic analysis under high-temperature: a proof of concept for postmortem interval estimation

**DOI:** 10.3389/fmed.2025.1661063

**Published:** 2025-09-11

**Authors:** Yinyu Chen, Gaolin Zheng, Xinyan Yang, Peng Zhang, Qianyun Nie

**Affiliations:** ^1^Key Laboratory of Tropical Translational Medicine of Ministry of Education, Department of Pathology, College of Basic Medical Sciences, Hainan Medical University, Haikou, China; ^2^Key Laboratory of Tropical Translational Medicine of Ministry of Education, Department of Forensic Medicine, College of Basic Medical Sciences, Hainan Medical University, Haikou, China

**Keywords:** forensic pathology, estimation of postmortem interval, metabolomics, femoral muscle, high temperature

## Abstract

**Introduction:**

Accurate postmortem interval (PMI) estimation is a major challenge in forensic medicine, especially for the rapidly and highly putrefied bodies in tropical high-temperature areas. Despite numerous studies, there are still no reliable, objective methods to accurately estimate PMI for corpses found in a high-temperature environment.

**Methods:**

In the current research, an ultra-high-performance liquid chromatography-quadrupole time-of-flight mass spectrometry (UPLC/Q-TOF MS)-based metabolomics approach was employed for the analysis of the metabolic profile of rat femoral muscle tissue in a high-temperature environment at different postmortem intervals. Multivariate pattern recognition and pathway analyses were employed for the identification of differential metabolites.

**Results:**

This study identified 14 significantly changed metabolites and two altered metabolic pathways. Among them, L-threonine, histidyl-threonine, L-tryptophan, N6-acetyl- L-lysine, eicosapentaenoic acid, glycerol 3-phosphate, and creatine were selected as possible diagnostic biomarkers for PMI estimation. Their impact value and potential biological function in the organisation’s degradation process were the basis for selection.

**Discussion:**

This study demonstrated the feasibility of UPLC/Q-TOF MS-based metabolomics in femoral muscle tissue research and identified several differential metabolites that may provide forensic scientists with a helpful reference in PMI estimation in a high-temperature environment.

## 1 Introduction

In forensics, the postmortem interval (PMI) refers to the time of death or the time elapsed since death, which means the time interval between the examination of the corpse and the occurrence of death (1). PMI estimation is the most challenging task for forensic scientists in forensic practice. It holds great importance in investigating criminal cases, such as providing important clues for revealing the nature of the cases, determining the direction of the investigation, locking or excluding suspects, and delimiting the scope of the investigation (2–4). Due to the paramount significance of accurately estimating the PMI in forensic sciences and criminal investigations, numerous methods have been proposed for its accurate determination. These methods include observation of the early cadaver phenomena, cadaver biomechanics test, Fourier infrared spectroscopy test, magnetic resonance spectroscopy test, microbiology, and entomology and so on (5–12). However, due to their reliance on multiple contexts and environmental factors, the accuracy and precision of these methods decrease with increasing PMI, and special care should be taken when developing such estimates and their inherent problems and limitations need to be considered and addressed in practical applications. Forensic pathologists find it difficult to determine the accurate PMI based on the above methods, particularly for the rapidly and highly putrefied bodies in tropical high-temperature areas (13, 14). Considering these facts, a significant demand remains for a reliable, objective, and universal laboratory-based method that can accurately estimate the PMI.

Therefore, over the past few years, many researchers have attempted to discover new techniques to determine the exact PMI. Biochemical indicator tests, such as ion composition, nucleic acid degradation, and enzyme activity tests, have shown significant advantages in PMI estimation and have attracted attention within the forensic community (15–17). Among these, biochemical analysis of the vitreous humor is critical for inferring time of death and provides an accurate and practical method for PMI inference due to its unique anatomical location and less interference from external factors. By detecting specific metabolite changes in the vitreous, combined with modern assay techniques, the accuracy of PMI inference can be significantly improved. Thus, Cordeiro et al. (16) employed a combination of various statistical methods and concentrations of hypoxanthine, potassium, and urea in vitreous fluid samples from 331 cadavers that had suffered sudden and unexpected deaths to calculate the PMI. A study by Noshy (15) investigated the expression of apoptosis-related genes in the livers of mice immediately, 3 h, 6 h, 9 h, 12 h, 18 h, and 24 h postmortem, and used these changes to estimate PMI. Forensic scientists validate biochemical indicators for PMI estimation through stringent testing and method refinement. While these indicators face challenges in specificity and stability, they are essential for forensic investigations, providing critical insights into both natural and violent deaths. Continuous advancements in detection techniques aim to enhance their reliability and applicability in the forensic field. This is particularly true for the rapidly and highly putrefied bodies in high-temperature environments (1, 16). In addition to the physical, chemical, and biological methods, omics-based techniques have gained popularity in developing reliable laboratory-based methods to estimate PMI.

In biological systems, metabolites refer to low-molecular-weight compounds that are synthesized or degraded by cells or tissues during life processes. These endogenous biochemical substances broadly include amino acids, lipids, fatty acids, steroids, carbohydrates, and vitamins. The complete set of these small-molecule metabolites constitutes what is known as the metabolome (18). Metabolomics, which is dedicated to deriving an unbiased and comprehensive analysis of hundreds of small endogenous molecules (<1,000 Da) in biological samples, has been successfully applied in the forensic medicine field in recent years (19). It can be conducted in a targeted or untargeted manner to analyze specific categories of metabolites (such as amino acids or fatty acids) or particular metabolic pathways (such as COX/LOX-derived products in the arachidonic acid metabolism pathway), thereby reflecting the dynamic changes associated with physiological or pathological states (18). In forensic research, metabolomics has been widely applied for the estimation of PMI. Previous studies have shown that multiple categories of metabolites exhibit time-dependent changes during the process of decomposition, and may serve as potential biomarkers for PMI prediction (20). These metabolites mainly include energy metabolism products, amino acids, purine degradation products, organic acids, as well as lipid metabolites and glycerol (20). Their postmortem accumulation or depletion is driven by factors such as cellular disintegration, ongoing enzymatic reactions, or microbial involvement, and they exhibit modelable increasing or decreasing trends as the PMI extends (20). Therefore, integrating high-throughput metabolomics platforms with machine learning approaches holds promise for identifying highly time-sensitive and specific PMI predictors among these key metabolites, providing more objective and quantitative support for time of death estimation under complex environmental conditions. Cellular metabolism and degradation leading to changes in metabolites with time to death must be considered along with other variables such as temperature and cause of death, especially in cases of violence. Thus, recognition of these factors provides a valuable reference for identifying metabolic profiles and significantly changing metabolites for PMI estimation (19). At present, three analytical technologies, namely nuclear magnetic resonance (NMR) spectroscopy, gas chromatography-mass spectroscopy (GC-MS), and liquid chromatography-mass spectroscopy (LC-MS), are commonly utilized for non-targeted metabolomic mapping. Several studies have characterized the postmortem metabolites to identify PMI-related metabolites in liver tissue, spleen tissue, vitreous humor, etc. (21–26). However, the limitations of these previous studies include low NMR sensitivity, a limited dynamic range, and the failure to account for the high-temperature factor. Recent advancements in immunohistochemical techniques have significantly enhanced PMI estimation accuracy, particularly through quantitative analysis of protein degradation patterns (e.g., complement C3 and fibronectin degradation products) in musculoskeletal tissues. These protein-based methods now complement traditional metabolomic approaches, with the former providing structural degradation markers and the latter capturing dynamic biochemical changes (27).

Temperature is considered a crucial factor that significantly affects the corruption rate. Because of the high temperature throughout the year in tropical regions, including Hainan province in China, the corpses decay rapidly, which makes it more difficult to accurately estimate PMI using the above biological samples (8, 17). The skeletal muscle tissue is the most accessible source for forensic examination. As the most abundant tissue type in the human body, skeletal muscle is not only readily accessible for sampling but also relatively well-preserved due to protection by the skin (28). Compared to internal organs and neural tissues, postmortem changes in skeletal muscle occur later; however, its rate of decomposition is significantly faster than that of cartilage and bone (29). These characteristics make muscle tissue well-suited for routine analysis in forensic laboratories and capable of effectively reflecting postmortem biological processes, thereby making it an ideal sample for PMI research. Moreover, skeletal muscle tissue exhibits distinct advantages in the metabolomics analysis for estimating PMI. These include availability in copious amounts, ease and speed of procurement, a slower rate of putrefaction than other biological tissues, and low susceptibility to environmental factors or circumstances of death (30, 31). Thus, skeletal muscle tissue may be an optimal specimen to develop biomarkers to estimate PMI in tropical high-temperature areas. While skeletal muscle tissue offers these advantages, it is important to consider the variable of external temperature, which can significantly affect the decomposition process. This factor is particularly relevant in human cases due to the larger body mass and varying thermal properties, as opposed to smaller animals where the effect may be negligible. The location, depth and isolation of the sample can introduce variability in the rate of decomposition, highlighting the need for minimally invasive sampling techniques that preserve the integrity and biological activity of the muscle tissue. Such measures ensure the accuracy of the sample and contribute to obtaining reliable experimental data for PMI estimation in tropical high temperature regions. Therefore, under high-temperature exposure, metabolic activity and degradation processes in muscle tissue are significantly accelerated. We hypothesize that specific metabolites or metabolic pathways exhibit regular, time-dependent, and reproducible changes during the progression of PMI, which can be utilized to establish predictive models for PMI estimation.

In the current investigation, an ultra-high-performance liquid chromatography quadrupole-time-of-flight mass spectrometry (UPLC/Q-TOF MS)-based metabolomics was employed for the analysis of the metabolic profile of rat femoral muscle tissue in a high-temperature environment at different postmortem intervals. Multivariate pattern recognition and pathway analyses were also carefully employed to excavate differential metabolites. This study aimed to develop new techniques for PMI estimation using postmortem metabolite changes under a high-temperature environment. The findings confirmed the feasibility of UPLC/Q-TOF MS-based metabolomics analysis in femoral muscle tissue research. It also identified several differential metabolites that may provide forensic scientists with a helpful reference in PMI estimation in a high-temperature environment.

## 2 Materials and methods

### 2.1 Chemicals and materials

Chemicals utilized in this experiment, including formic acid, methanol, ammonium acetate, ammonium fluoride, and acetonitrile, were of high-performance liquid chromatography grade and were provided by Sigma-Aldrich (St. Louis, MO, United States). The Milli-Q water purification system (Millipore, Bedford, MA, United States) was employed to prepare ultrapure water for this experiment.

### 2.2 Animals and ethics statement

The study utilized 32 adult male Sprague–Dawley rats supplied by the laboratory animal center of Chongqing Medical University (Chongqing, China). The rats had an average age of 8 weeks and were weighed approximately 250–300 g. The animals were kept under standard experimental laboratory conditions of 21–24 °C, 40–70% relative humidity, and a 12 h light/dark cycle. The animals were subjected to an acclimatization period of 1 week in the laboratory environment before the commencement of the experiment. All animal procedures conducted in this study strictly adhered to the guidelines provided by the Guide for the Care and Use of Laboratory Animals, and the approval was granted by the Ethics Committee (HYLL-2021-303).

The animals were sacrificed through cervical dislocation and placed in an artificial climate chamber (Shanghai STIK Co., Ltd., China). The conditions set in the chamber included relative humidity of 45–55%, a 12 h light–dark cycle, and a temperature of 35 °C. Afterward, the rats were classified randomly into four groups (*n* = 8 rats/group): control, 1 d, 2 d, and 3 d groups.

### 2.3 Sample collection and preparation

The femoral muscle tissues were rapidly removed from the quadriceps femoris of the hind limbs at the time points of 1, 2, and 3 days after death and rinsed with ice-cold normal saline to eliminate any adhesions. Liquid nitrogen was utilized to freeze the muscle tissues temporarily, which were subsequently kept at a temperature of −80 °C until further UPLC/Q-TOF MS-based metabolomic analysis. The femoral muscle tissues of the control animals were removed immediately after the animals were sacrificed. A 200 mg frozen muscle tissue sample was thawed on ice and transferred in a 1 mL centrifuge tube with 800 μL acetonitrile. The centrifuge tube was placed on ice, and the muscle tissue was pulverized using a magnetic bead grinder. Subsequently, the samples were centrifuged at 12,000 rpm for 5 min at 4 °C. After centrifugation, 600 μL of the supernatant was aspirated into a new centrifuge tube using a thin-film filter pipettor (0.22 μm, nylon 66), concentrated and dried in a vacuum concentrator. Afterward, the samples were redissolved in a solvent consisting of 100 μL acetonitrile/water (1:1, v/v). From each sample, 2 μL was injected into the UPLC system. To observe the stability and reproducibility of the instrument analysis, 20 μL of all the samples were mixed to create quality control (QC) samples and were subsequently analyzed alongside the other samples.

### 2.4 UPLC/Q-TOF/MS analysis

The Exion ultra-high performance liquid chromatography system coupled with a triple-quadrupole time-of-flight 6600 mass spectrometry (Q-TOF/MS; AB SCIEX, Massachusetts, United States) was utilized for the metabolomic analysis of femoral muscle. MS/MS analysis on non-target metabolites provided a qualitative and quantitative table of metabolites by examining the mass-to-charge ratios of parent and daughter ions, matched with the database. The procedures for metabolomic analysis have been published recently in detail (32, 33). The compounds in the samples were isolated utilizing an ultra-high performance liquid chromatography system on an ACQUITY UPLC HSS T3 column (1.8 μm, 2.1 mm × 10 mm, Waters) maintained at 25 °C. An aqueous solution of 0.1% formic acid as mobile phase A and acetonitrile plus 0.1% formic acid as mobile phase B were utilized for gradient elution. The flow rate in the analysis was set at 0.3 mL/min. The elution gradient followed the following pattern: 0 to 1.5 min, 1% B; 1.5 to 11.5 min, linearly increased to 99% B; 11.5 to 15 min, 99% B; 15 to 15.1 min, 1% B, followed by equilibration to 19 min. After passing through the UPLC system, the mass spectrometric analysis was performed on the liquid chromatography eluent with the Triple-TOF 6600 system in both positive and negative ion modes using the Turbo V ESI ion source. The TripleTOF 6600 mass spectrometer facilitated the acquisition of MS/MS spectra on an information-dependent basis (IDA) during LC/MS experiments. The Analyst TF 1.7 software (AB Sciex) continuously evaluated full scan survey MS data, triggering MS/MS spectrum acquisition based on predefined criteria. The mass spectrometer was configured with the following parameters: ion-spray voltage: 5.5 kV (+) and −4.5 kV (−); nebulizer gas and turbo gas: 55 PSI; heater temperature: 600 °C; curtain gas: 25 PSI; collision energy: 10 V (+) and −10 V (−); and mass spectra range: 50–1,000 *m*/*z* in both positive and negative ion mode.

The experiment employed three technical replicates to ensure data reproducibility, and an internal standard mixture was used to normalize mass spectrometry responses. QC samples were interspersed throughout each batch to monitor and correct for batch effects. The QC samples were prepared by mixing equal aliquots of the supernatants from all of the samples to avoid intra-batch variability, as well as to evaluate the analytical quality of the UPLC/Q-TOF MS analysis system (32, 33). The QC samples were injected after every eight real samples. The relative standard deviations (RSD) were calculated to ensure the suitability and stability of the metabolomic analytical system. The internal standard, an isotopically labeled metabolite with a consistent concentration in QC samples, indicated system stability and data quality, with RSD ≤20% reflecting high stability. Deviation filtering based on RSD (coefficient of variation, CV) was performed, with % CVs for retention time and peak area <50% for each standard, regardless of fraction or matrix. Numerical simulation involved filling in half of the minimum value, and normalization was performed using the total ion current (TIC) of each sample. The repeatability and reliability of the analytical system was first assessed by the overlapped performance of the spectral peaks of the four within-run QC samples, and further evaluated by relative standard deviation (RSD%) of eight randomly picked ions from chromatographic peaks in the four within-run QC samples (34, 35).

### 2.5 Data processing and analysis

ProteoWizard was initially utilized to convert the raw data from the metabolomic analysis into mzXML files. Lipid-View (AB SCIEX, Massachusetts, United States) was employed to process the data for automated alignment, peak recognition, integration, normalization, correction, etc., before multivariate statistical analysis. First, after importing the data, chromatogram alignment is automatically performed to ensure that compound signals are correctly matched across samples; then peaks in the chromatograms are identified and integrated to quantify compounds; then data normalization is performed to eliminate experimental bias and improve data quality through baseline correction and noise removal. Before proceeding with multivariate statistical analysis, specific corrections were applied to the data to adjust for experimental variability and ensure the assumptions of statistical tests were met. The Paragon database search algorithm and FDR analysis in ProteinPilot facilitated peptide identification, with a 95% confidence interval (CI) set as the threshold for significance. After data acquisition, the dataset containing product ions, retention time, normalized ion intensities, and sample names was imported into SIMCA-P v14.0 (Umetrics, Umeå, Sweden) for multivariate pattern recognition analysis. Log transformation and UV scaling were applied to eliminate dimensional differences and enhance model stability. Subsequently, automatic modeling analysis was performed to provide a basis for subsequent biological interpretation. Principal component analysis (PCA) and orthogonal partial least squares discriminant analysis (OPLS-DA) with autoscaling and mean centering were used to visualize differences between experimental and control group samples, identifying metabolic profile changes induced by high temperature. Three parameters (*R*^2^*X*, *R*^2^*Y*, and *Q*^2^) were utilized to illustrate the validity and robustness of the OPLS-DA model. Model quality was evaluated using 7-fold cross-validation, and model validity was assessed by *R*^2^*Y* (the model’s explained variance of the classification variable *Y*) and *Q*^2^ (the model’s predictive ability). To ensure model robustness and prevent overfitting, a 200-times permutation test was conducted. The *Q*^2^-intercept was <0, confirming the model’s statistical validity. Distinct variables were found through the variable importance in projection (VIP) values derived from the OPLS-DA model. Afterward, Mass Profiler Professional (Agilent Technologies, United States) was employed to perform statistical analysis and calculate fold change (FC) for variables that exhibited VIP values exceeding 1. Significantly changed variables were identified based on the following limitations: (1) *p*-value <0.05; (2) FC values between the experimental and control group >1.5 or <0.67; and (3) VIP >1. The potential differential metabolites were screened by querying their exact accurate *m*/*z*, retention times, and MS/MS spectra in the online databases, which included HMDB^1^ and METLIN.^2^ Pathways analysis and visualization of the significantly changed metabolites were conducted utilizing the online MetaboAnalyst tool^3^ based on the Kyoto Encyclopedia of Genes and Genomes database source. Clustering analysis of the significantly changed metabolites was performed to visualize the differences of metabolite signatures between the groups.

Data analysis was conducted utilizing SPSS 21.0 (IBM, Armonk, NY, United States). The Shapiro–Wilk test assessed the normality of distributions. Considerable variations among various groups were assessed through a one-way analysis of variance (ANOVA), followed by *post hoc* comparisons using Tukey’s honest significant difference test. Significance was considered achieved when the two-tailed *p*-value was <0.05.

## 3 Results

### 3.1 Femoral muscle metabolic profile based on UPLC/Q-TOF MS

**Figure 1:**
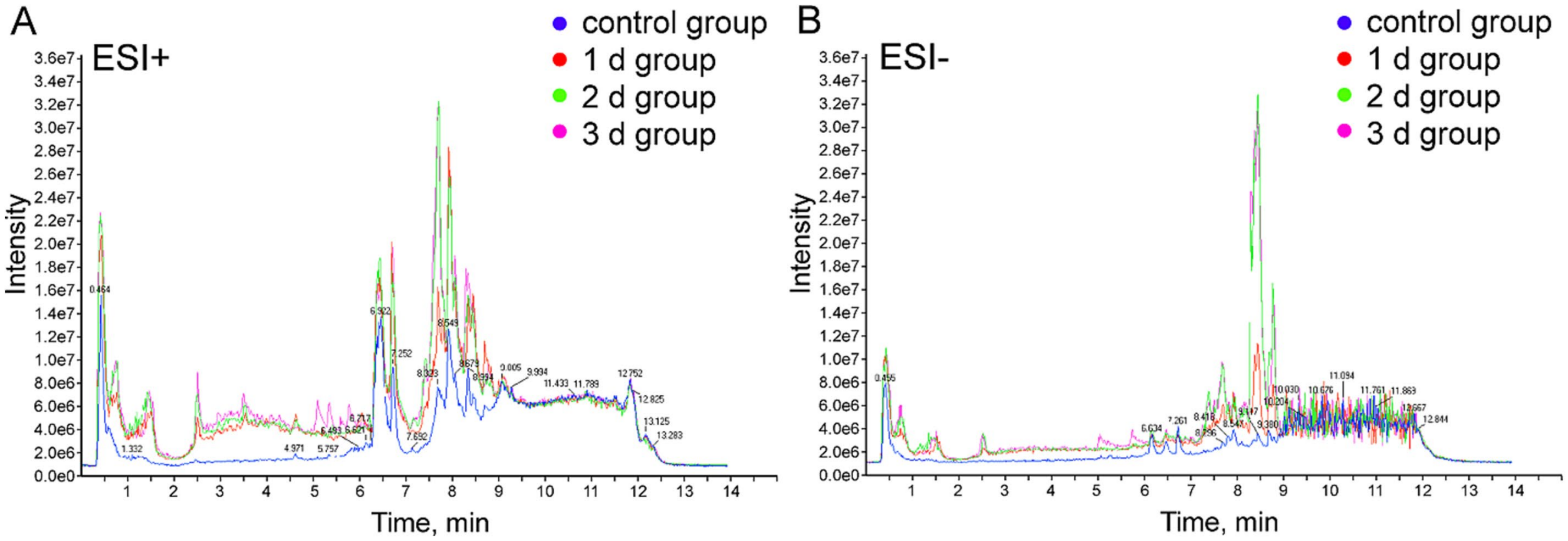
Representative TIC of femoral muscle tissue samples from the control and the experimental groups. **(A)** Positive ion mode (ESI^+^). **(B)** Negative ion mode (ESI^−^).

In the current research, metabolomics analysis was carried out utilizing UPLC/Q-TOF MS technology in both positive and negative ion scan modes to identify potential biomarkers in femoral muscle tissue to enhance the accuracy of PMI estimation in a high-temperature environment. The total ion current (TIC) chromatograms of femoral muscle tissue samples obtained from both groups in positive and negative models are illustrated in Figure 1. Considerable variations were observed between control and experimental groups, indicating that the metabolites of femoral muscle tissue in a high-temperature environment significantly changed with the PMI. Meanwhile, RSD values and TIC chromatograms of QC samples in the metabolomic analysis system were suitable and stable, which confirmed the feasibility of the UPLC/Q-TOF MS technology in further analyses (Supplementary Figure S1 and Supplementary Table S1).

### 3.2 Multivariate analysis

**Figure 2:**
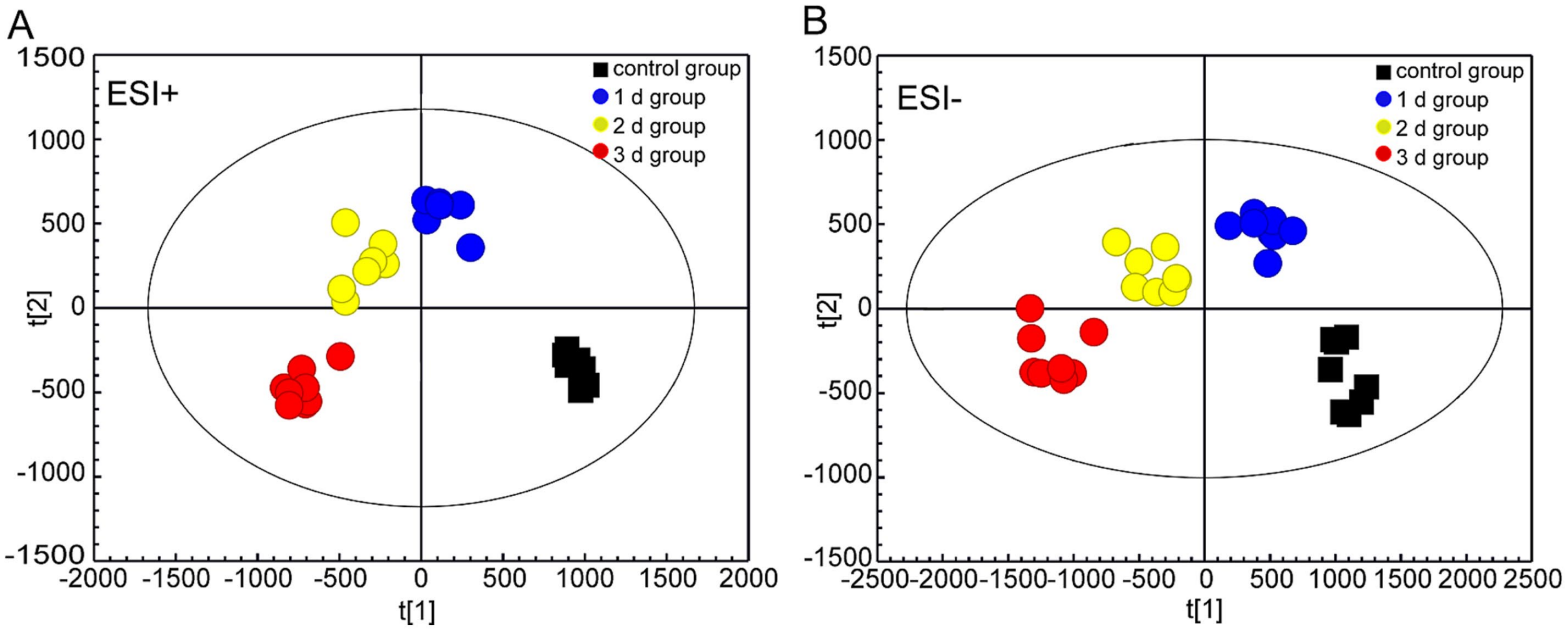
UPLC/Q-TOF MS-based OPLS-DA analyses of the eight femoral muscle samples collected from the 1 d, 2 d, 3 d, and control groups under positive and negative electrospray ionization modes. **(A)** Positive ion mode (ESI^+^). **(B)** Negative ion mode (ESI^−^).

**Figure 3:**
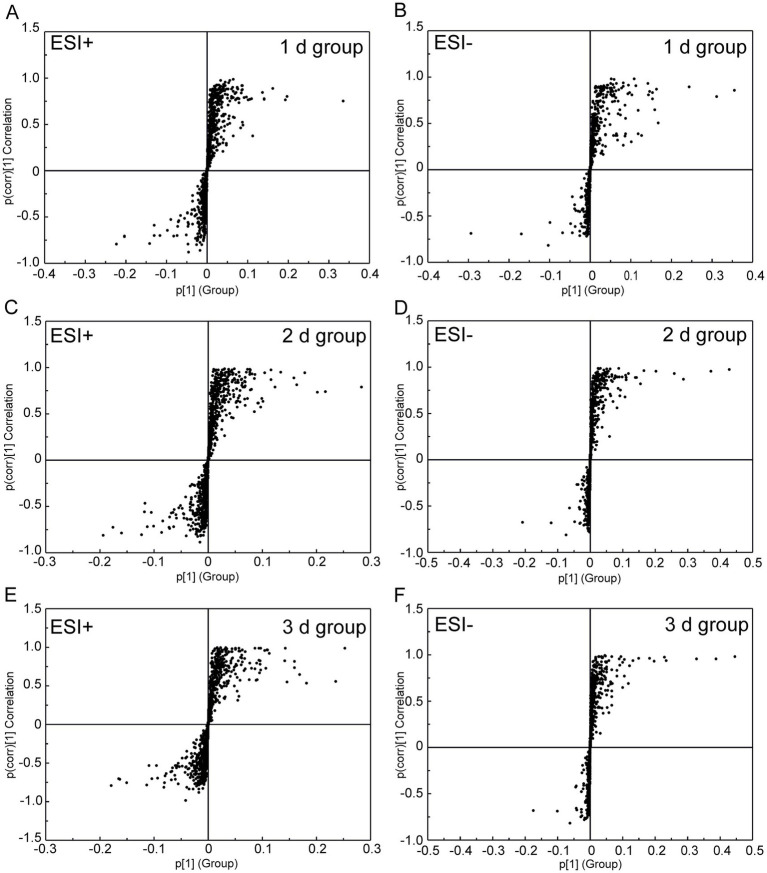
S-plot from the OPLS-DA analysis of UPLC/Q-TOF MS data from the 1 d, 2 d, 3 d, and control groups under negative and positive electrospray ionization modes. **(A,B)** Score plots of the 1 d group (24 ± 2 h, *n* = 8). **(C,D)** Score plots of the 2 d group (48 ± 2 h, *n* = 8). **(E,F)** Score plots of the 3 d group (72 ± 2 h, *n* = 8). All analyses included three technical replicates, with the time error range derived from standardized postmortem interval controls (±2 h).

**Figure 4:**
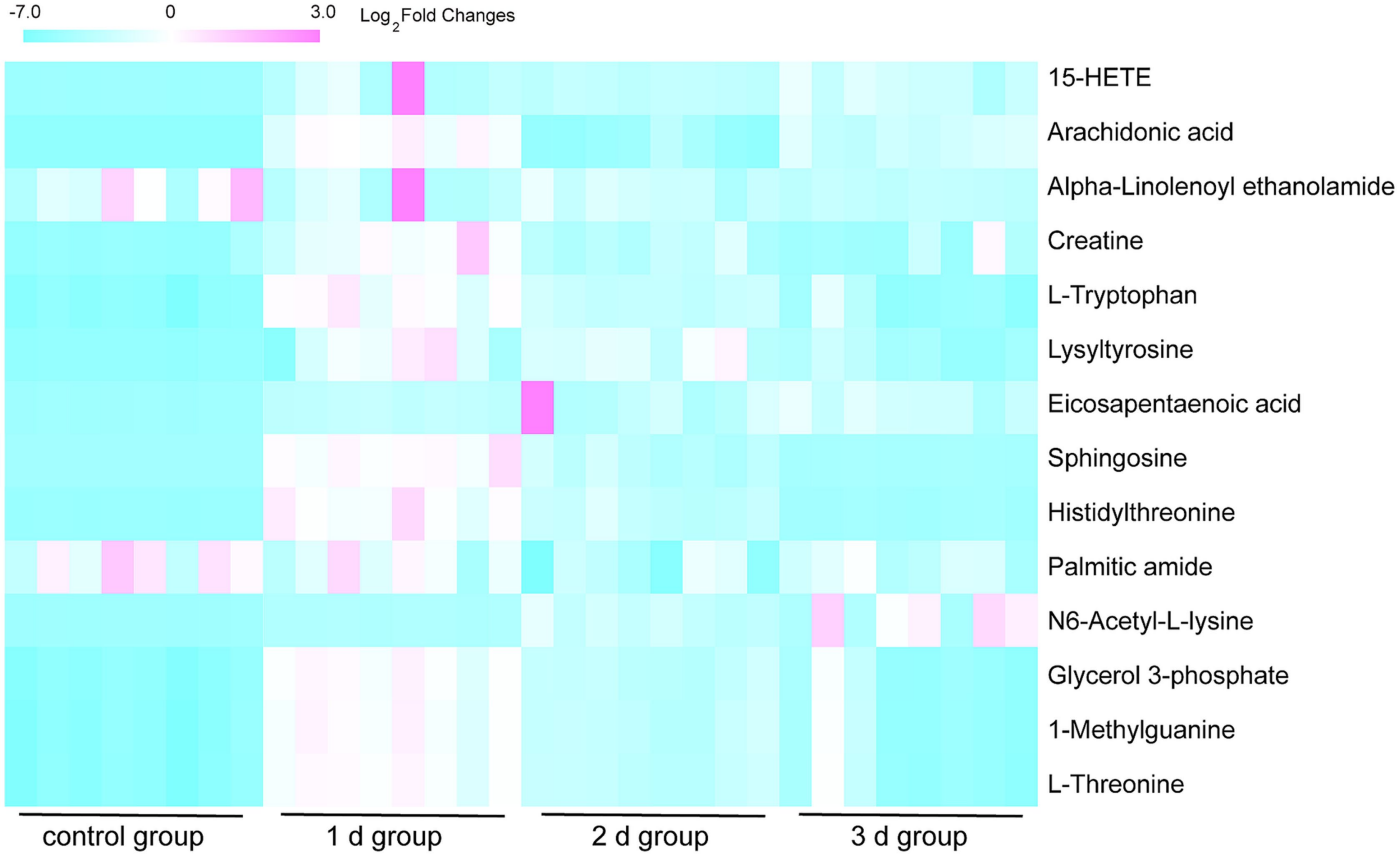
Heat map of hierarchical clustering of the 14 differential metabolites in the eight femoral muscle tissue samples from the 1 d, 2 d, 3 d, and control groups. Red blocks demonstrate upregulation, and blue demonstrate downregulation. The outcome demonstrates a robust metabolic variation between the experimental and control groups.

By employing UPLC/Q-TOF MS metabolomics analysis on femoral muscle tissue samples obtained from both high-temperature-exposed and control rats, 256 metabolite components were detected. These components collectively constituted the “metabolic profile” of the femoral muscle tissue throughout the degradation process following death. The metabolites were subsequently subjected to the multivariate analysis following normalization and alignment. PCA analysis was initially conducted to determine whether the experimental groups could be distinguished from the control group. The outcomes revealed that the femoral muscle tissue samples acquired from the experimental groups were distinguished from that of the control in negative and positive ion modes. These findings illustrate that considerable femoral muscle metabolic changes occurred in the rats following exposure to high temperature (Supplementary Figure S2). To get a clearer picture of the variables that differed between groups, supervised pattern recognition was performed using OPLS-DA analysis. The OPLS-DA score plots demonstrated clear segregation of both groups (Figure 2; ESI^+^: *R*^2^*X* = 0.673, *R*^2^*Y* = 0.956, *Q*^2^ = 0.896; ESI^−^: *R*^2^*X* = 0.467, *R*^2^*Y* = 0.979, *Q*^2^ = 0.905). The outcomes revealed both the robust metabolic alterations in femoral muscle tissue associated with the PMI in a high-temperature environment and the strong explanatory power of the data, along with the good predictive ability of the model. The results of permutation tests further confirmed the reliability and validity of the constructed OPLS-DA model [Supplementary Figure S3; ESI^+^: *R*^2^ = (0.0, 0.6), *Q*^2^ = (0.0, −0.633), ESI^−^: *R*^2^ = (0.0, 0.667), *Q*^2^ = (0.0, −0.546)]. Moreover, the S-plot from OPLS-DA analysis demonstrated that fourteen considerably altered metabolites with VIP >1, FC >1.5 or <0.67, and *p* < 0.05 were responsible for the discrimination in the score plots and were regarded as potential biomarkers (Figure 3 and Table 1). When these 14 significantly changed metabolites were subjected to cluster analysis to analyze the differential metabolite signatures in the experimental groups, a clear separation between both groups was revealed. The outcome of the study was visualized through a heat map (Figure 4).

**Table 1:** Differentially changed metabolites in the femoral muscle samples of the 1 d, 2 d and 3 d groups after death.

No.	Metabolite	Ion (*m*/*z*)	Retention time (min)	Fold change			ESI mode
				1 d	2 d	3 d	
1	L-Threonine	120.0806	1.68	1.40	0.29	0.23	ESI^+^
2	1-Methylguanine	166.0868	1.68	0.67	0.30	0.24	ESI^+^
3	Glycerol 3-phosphate	167.0896	1.56	0.62	0.27	0.21	ESI^+^
4	N6-Acetyl-L-lysine	188.0710	2.69	1.7	4.76	7.89	ESI^+^
5	Palmitic amide	256.2634	8.36	5.62	6.70	1.31	ESI^+^
6	Histidylthreonine	257.1132	1.57	1.45	0.04	0.01	ESI^+^
7	Sphingosine	300.2899	8.92	0.27	0.07	0.01	ESI^+^
8	Eicosapentaenoic acid	303.2338	7.98	0.08	0.05	0.85	ESI^+^
9	Lysyltyrosine	310.1761	2.60	0.99	0.09	0.04	ESI^+^
10	L-Tryptophan	205.0980	2.65	1.67	0.27	0.19	ESI^+^
11	Creatine	130.0873	1.00	0.45	0.17	0.13	ESI^−^
12	Alpha-Linolenoyl ethanolamide	320.2314	7.99	0.80	0.06	0.05	ESI^−^
13	Arachidonic acid	303.2323	9.04	0.66	0.38	0.13	ESI^−^
14	15-HETE	319.2277	8.01	0.81	0.71	0.07	ESI^−^

### 3.3 Differential metabolite analysis

Comparative analysis revealed differential trends in the metabolite levels in femoral muscle tissues after death (Figure 4). Methylguanine, glycerol 3-phosphate, sphingosine, lysyl-tyrosine, creatine, α-linoleoethanolamide, arachidonic acid, and 15-hydroxyeicosatetraenoic acid (15-HETE) levels decreased with the PMI, whereas L-threonine, histidine, and L-tryptophan levels elevated on day 1 and then reduced on days 2 and 3 after death; N6-acetyl-L-lysine level increased in all of three experimental groups of days 1, 2, and 3; Palmitic amide level increased on days 1 and 2 and then decreased on day 3 after death; and eicosapentaenoic acid (EPA) level decreased in the 1 d and 2 d groups and then elevated in the 3 d group. Changes in the metabolite levels may be attributed to their metabolic pathways after death. Overall, with the extension of PMI, the metabolites showed downward trends.

### 3.4 Metabolic pathway analysis

**Figure 5:**
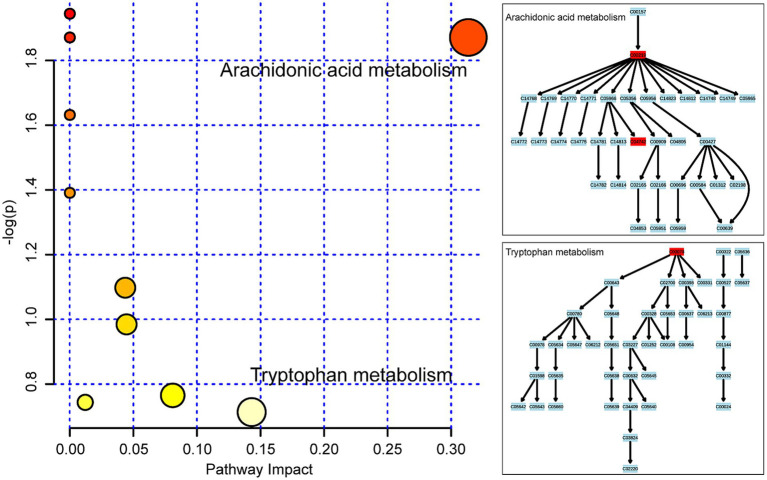
Metabolic pathway analysis of the 14 considerably changed metabolites in femoral muscle. Ten metabolic pathways were disrupted following high-temperature exposure and are demonstrated as circles and arranged as per the scores based on topology (x-axis) (y-axis) and enrichment analysis.

To determine the relevant pathways involved in the molecular mechanisms of high-temperature exposure, an analysis of the metabolomics pathways was conducted for the identified metabolites that exhibited significant variation. This analysis was performed using the online MetaboAnalyst tool. The outcome of the study identified 10 metabolic pathways that indicated increased levels of changes after exposure to high temperatures and are illustrated in the form of circles in Figure 5. Among them, changes in the impact values in two metabolic pathways, arachidonic acid and tryptophan metabolism, are noteworthy, suggesting that these pathways are affected to some extent during metabolism. Among the metabolites, arachidonic acid is from arachidonic acid metabolism, and 15-HETE and L-tryptophan are from tryptophan metabolism.

## 4 Discussion

Accurate PMI estimation is a major challenge in forensic medicine, especially for the rapidly and highly putrefied bodies found in tropical high-temperature areas. In meteorology, when a dry and hot southwest wind and subtropical high pressure persist over a long period, a temperature of ≥35 °C is considered “high-temperature weather.” If this temperature exceeds 35 °C for multiple consecutive days, it is called “heat wave weather.” The average temperature in Hainan province, China, is 16 °C, and it can reach as high as 30–35 °C as early as March. Thus, despite many studies, there are still no reliable, objective methods to estimate PMI accurately for the highly decayed corpses found in the high-temperature environment (36–39). The skeletal muscle can be rapidly and easily obtained in forensic examination and may be an optimal tissue to identify biomarkers to estimate the PMI in tropical high-temperature areas. Compared to conventional morphological observations and biochemical tests, metabolomics offers more information by detecting metabolites in biological samples. This direct association between the phenotype and PMI enables some of these metabolites to potentially function as indicators (18, 40–42). The objective of this research was to acquire the metabolic profiles of the femoral muscle in rats following exposure to high temperatures and identify potential biomarkers within the femoral muscle, thus enhancing the accuracy of PMI estimation, specifically in high-temperature environments.

The UPLC/Q-TOF MS-based metabolomics approach was performed for the identification of differential femoral muscle metabolites. In total, 14 metabolites were identified through the multivariate statistical analysis that showed significant changes in femoral muscle following exposure to high temperature. Metabolic pathway analysis of these metabolites revealed disruption of 10 metabolic pathways. By comparing their impact values, arachidonic and tryptophan metabolic pathways were identified as remarkably affected, with arachidonic acid, 15-HETE, and L-tryptophan as the major components with differential levels. Moreover, hierarchical cluster analysis was utilized for the analysis of the selected differential metabolites. The changes in the metabolites in rat femoral muscle with PMI could be found. Thus, the comparative analysis revealed that the metabolites showed different trends in femoral muscle after death.

Arachidonic acid metabolism is a key lipid signaling pathway involving enzymatic conversion of arachidonic acid by cyclooxygenases (COX-1/2) and lipoxygenases (LOX-5/12/15) into a range of bioactive eicosanoids (43). These include prostaglandins (PGs), thromboxanes (TXs), and hydroxyeicosatetraenoic acids (HETEs), which are known to modulate inflammation, vascular tone, and immune responses (44). In the present study, we observed a progressive decline in arachidonic acid and its downstream product 15-HETE with increasing PMI, suggesting time-dependent degradation or enzymatic inactivation under postmortem conditions. The decrease in these metabolites may reflect not only the cessation of enzymatic activity after death, but also potential thermal degradation of polyunsaturated fatty acids under high-temperature exposure. While inflammation-related activation of COX/LOX pathways may occur in agonal or traumatic deaths, the monotonic decline observed in our study supports a postmortem origin rather than a perimortem spike. Further, the reduction of LOX-derived 15-HETE—a compound susceptible to oxidative decay—may also indicate enhanced lipid peroxidation or enzymatic degradation in the early stages of decomposition. These findings highlight the importance of integrating biochemical pathway dynamics with PMI estimation models, while also considering confounding factors such as enzymatic decay, microbial activity, and environmental temperature.

Tryptophan metabolism is a multifaceted pathway involved in immune regulation, neurotransmitter synthesis, and redox homeostasis, primarily proceeding via the kynurenine, serotonin, and indole routes (45). In the current investigation, levels of amino acids were considerably elevated in the 1 d group and decreased in the 2 d and 3 d groups compared with controls. As protein synthesis ceases after death, the increase in these amino acids may be related to the cessation of protein synthesis and degradation of proteins after death (46, 47). Furthermore, these amino acids rapidly decrease in femoral muscle after death, which may be associated with the autolysis and spoilage of proteins after death. These findings suggest that although the autolysis and spoilage of muscle tissue occurred more slowly compared with other tissues, the rate can be accelerated in the high-temperature environment. It is noteworthy that in addition to temperature effects, the interplay of various pre-mortem conditions, such as the duration of oxygen deprivation and the period of distress prior to death, adds a layer of complexity to the post-mortem biochemical timeline. These factors can modulate the rate at which amino acids fluctuate and, in turn, affect the correlation between these biochemical markers and PMI. Understanding this variability enriches our interpretation of the biochemical processes at play and provides a more nuanced approach to estimating PMI in different scenarios.

Another selected metabolite in the study was N6-acetyl-L-lysine, which was upregulated in the high-temperature-exposure groups relative to the control animals. Pesko et al. (48) found lysine as one of the most promising biomarker candidates for PMI estimation. Histones contain a large amount of lysine, which could produce N6-acetyl-L-lysine under the action of n-acyl peptide hydrolase as a lysine derivative. The findings of this research are consistent with those of the previous relevant studies (49). It was speculated that the upregulation of N6-acetyl-L-lysine with PMI is related to the autolysis and histone decomposition of femoral muscle after death, thus indicating that N6-acetyl-L-lysine could be a potential marker for PMI estimation.

In the current investigation, EPA was the only metabolite that was considerably decreased in the femoral muscle of the 1 d and 2 d groups but increased in the 3 d group relative to that in the controls. EPA is an important polyunsaturated fatty acid and participates in numerous biological processes (50). Although a significant decrease in EPA was observed within 1–2 days postmortem followed by a rebound on day 3 (*p* < 0.05), the underlying mechanisms require further evidence. Current data suggest that this phenomenon may involve two competing processes: an early phase (1–2 days) characterized by phospholipase A2-mediated degradation of membrane phospholipids leading to reduced EPA release, and a later phase (day 3) where diminished microbial β-oxidation combined with adipose tissue autolysis may contribute to secondary EPA release. However, these hypotheses need to be validated through integrated targeted lipidomics (e.g., monitoring sn-2 position-specific hydrolysis) and microbial community sequencing (e.g., changes in Proteobacteria abundance). In addition, glycerol 3-phosphate and creatine were the other two selected metabolites in this study, which were down regulated in the high temperature-exposure group than in the control animals. Glycerol 3-phosphate and creatine play significant roles in energy metabolism. The decrease in glycerol 3-phosphate and creatine may be related to energy consumption in muscle tissue and the termination of energy generation after death (51, 52).

In summary, a UPLC/Q-TOF MS-based metabolomics approach was utilized in combination with multivariate pattern recognition and pathway analysis to screen PMI-related femoral muscle metabolites of rats after high-temperature exposure. In total, 14 considerably changed metabolites and two disrupted metabolic pathways were detected in this research. Among them, seven metabolites, namely L-threonine, histidyl-threonine, L-tryptophan, N6-acetyl-L-lysine, EPA, glycerol 3-phosphate, and creatine, were selected as possible diagnostic biomarkers for PMI estimation as these might be involved in the process of autolysis and corruption after death. Although the exact metabolite list in this study is not entirely overlapping with prior PMI-related metabolomics reports, several of the compounds identified—such as creatine, amino acids (e.g., L-tryptophan, histidine), and nucleotide derivatives (e.g., hypoxanthine, glycerol-3-phosphate)—have been previously associated with postmortem interval progression in other biological matrices, including liver, spleen, and vitreous humor (52–54). This partial convergence supports the biological plausibility and forensic relevance of the observed metabolite changes. The biological rationale for these alterations is also consistent with known postmortem biochemical processes. For instance, the early rise in amino acids likely results from autolytic proteolysis, whereas the decline in energy-related metabolites such as creatine and ATP may reflect cessation of mitochondrial activity (55). Lipid mediators like arachidonic acid and 15-HETE are known to be susceptible to thermal degradation and oxidative decay, which explains their decreasing trends under high-temperature conditions. On the other hand, stable molecules like N6-acetyl-L-lysine showed persistent accumulation, suggesting potential use as longer-term PMI markers. Traditional postmortem interval estimation methods, such as algor mortis, rigor mortis, and morphological scoring systems, often lose accuracy under high-temperature or advanced decomposition conditions. In contrast, muscle tissue—particularly the quadriceps femoris—exhibits relative biochemical stability and slower degradation rates, making it a promising candidate for molecular-level analyses. The consistent trends observed in metabolite alterations provide a foundation for future time-dependent regression modeling. These findings indicate that metabolomic profiling of femoral muscle may offer valuable insights into PMI-related biochemical changes, providing a potential basis for future model development and forensic applications, especially under high-temperature exposure.

However, there are a few limitations to this study. First, only three time points were studied, and the dynamic evolvement of the metabolites obtained after 3 days was not evaluated. Future studies would focus on an extended observation period that may help obtain a deeper comprehension of the identified metabolites after death in a high-temperature environment. To refine this critical dimension, we are planning a systematic follow-up study incorporating a more granular temporal gradient. Specifically, high-frequency sampling every 12 h will be conducted within the 0–72 h window to capture early disturbances in energy metabolism, while daily sampling from days 4 to 7 will be performed to monitor the temporal dynamics of membrane stability-related metabolites. Second, these study results are based on a rat model and must be validated in a real-life scenario. There are three key differences in muscle metabolomics between rodents and humans: (1) Differences in basal metabolic rate—rats exhibit a metabolic rate approximately seven times higher than that of humans—which may accelerate metabolite turnover; (2) Variations in muscle fiber composition, with rats having a higher proportion of type IIb fast-twitch fibers, which can influence the degradation kinetics of specific metabolites such as creatine and carnitine; (3) Distinct thermoregulatory mechanisms—humans primarily rely on sweating, whereas rats regulate temperature through panting—may result in divergent postmortem cooling curves. These factors indeed affect metabolite stability, particularly for thermolabile compounds such as ATP and glutathione. Based on these observations, our subsequent research will implement a stepwise translational validation strategy. In the first phase, a human–rat paired sample comparison study will be conducted to identify conserved cross-species metabolic markers. In the second phase, a multicenter human cohort will be used to develop machine learning correction models that integrate key forensic variables, including BMI and environmental conditions. To address practical casework scenarios, future investigations will systematically examine exogenous factors such as clothing insulation and individual muscle mass differences, and develop temperature compensation algorithms based on the Arrhenius equation. Extensive investigations are essential to identify efficient biomarkers that accurately determine the PMI. In addition, by controlling for the variable of high temperature in this study, we were able to more accurately observe and quantify the effect of high temperature on the rate and pattern of carcass decomposition, thus reducing confounding by other factors. Experiments conducted under controlled conditions are more likely to be replicated by other researchers, which helps to verify the reliability and reproducibility of the results. However, cadaver decomposition in the real world is affected by a variety of factors, including humidity, microorganisms and animal interference. Experiments that control for high temperatures may not adequately account for other ecological factors that also have a significant effect on cadaver decomposition in natural environments. Variations in humidity may significantly alter the degradation trajectories of amino acids such as proline and γ-aminobutyric acid by modulating the rate of tissue hydrolysis. Heterogeneous enzymatic activity from soil microbiota can interfere with key PMI markers. Additionally, insect activity—particularly from Dipteran larvae—may induce mechanical disruption of muscle tissue and artificial loss of metabolites. To address these challenges, future experiments will incorporate a multifactorial environmental simulation platform. This platform will integrate controlled humidity gradients (30–90% relative humidity), standardized inoculation with soil-derived microbial communities, and exposure to arthropods. Using an orthogonal experimental design, the relative contributions of each environmental variable to the metabolite feature matrix will be systematically evaluated. These data will inform the development of PMI prediction models with environmental parameter correction, aiming to enhance model applicability under complex field conditions.

The application of muscle-based metabolomics in forensic workflows requires the standardization of sampling protocols, validation using human tissues, and integration with environmental data such as temperature and humidity. Therefore, the findings of the present study should be regarded as a proof of concept. Future research should aim to incorporate larger sample sizes, broader postmortem interval (PMI) ranges, and multivariate modeling approaches to enhance predictive accuracy and forensic applicability.

## Data Availability

The raw data supporting the conclusions of this article will be made available by the authors, without undue reservation.

## Footnotes

^1^ http://www.hmdb.ca/

^2^ https://metlin.scripps.edu

^3^ http://www.metaboanalyst.ca
