# Supplementary material for: UPLC/Q-TOF MS-based femoral muscle metabolomic analysis under high-temperature: a proof of concept for postmortem interval estimation
Source: Front Med (Lausanne). 2025 Sep 11;12:1661063. doi: 10.3389/fmed.2025.1661063 (PMC12460302; doi:10.3389/fmed.2025.1661063)
Supplement: Supplementary file 1 [file Data_Sheet_1.doc]

**Supplementary Material**

**UPLC/Q-TOF MS-based Femoral Muscle Metabonomic Analysis Under High-Temperature: A Proof of Concept for Postmortem Interval Estimation**

Yinyu Chen^a,#^, Gaolin Zheng^b,#^, Xinyan Yang^b,#^, Peng Zhang^b,*^, Qianyun Nie^a,**^

^a^ Department of Pathology & Key Laboratory of Tropical Translational Medicine of Ministry of Education, College of Basic Medical Sciences, Hainan Medical University, Xueyuan Road 3#, Longhuaqu, Haikou, 571199, Hainan, China.

^b^ Department of Forensic Medicine & Key Laboratory of Tropical Translational Medicine of Ministry of Education, College of Basic Medical Sciences, Hainan Medical University, Xueyuan Road 3#, Longhuaqu, Haikou, 571199, Hainan, China.

^#^ These authors contributed equally to this work.

^*^ Corresponding author: Peng Zhang, Department of Forensic Medicine & Key Laboratory of Tropical Translational Medicine of Ministry of Education, College of Basic Medical Sciences, Hainan Medical University, Xueyuan Road 3#, Longhuaqu, Haikou, 571199, Hainan, China. E-mail: 972421821@qq.com.

^**^ Corresponding author: Qianyun Nie, Department of Pathology & Key Laboratory of Tropical Translational Medicine of Ministry of Education, College of Basic Medical Sciences, Hainan Medical University, Xueyuan Road 3#, Longhuaqu, Haikou, 571199, Hainan, China. E-mail: nieqianyun0606@126.com.

Table S1. The stability and reproducibility of the UPLC/Q-TOF MS analytical system was assessed by eight ions of the QC samples in both positive and negative ESI mode.

| Peaks No. | ESI+ | | |  | ESI- | | |
| --- | --- | --- | --- | --- | --- | --- | --- |
|  | Retention time (min) | m/z | RSD (%) |  | Retention time (min) | m/z | RSD (%) |
| 1 | 1.369 | 214.1079 | 2.25 |  | 1.364 | 275.0241 | 3.24 |
| 2 | 2.697 | 118.0865 | 3.23 |  | 2.223 | 273.0087 | 2.59 |
| 3 | 6.898 | 150.0259 | 4.24 |  | 4.748 | 363.0184 | 5.12 |
| 4 | 7.246 | 101.0958 | 2.16 |  | 5.199 | 274.0113 | 4.28 |
| 5 | 8.255 | 302.1423 | 4.53 |  | 6.886 | 138.0202 | 1.24 |
| 6 | 8.542 | 87.0439 | 3.24 |  | 7.334 | 187.1346 | 5.04 |
| 7 | 9.797 | 126.0664 | 5.12 |  | 14.152 | 239.0943 | 3.25 |
| 8 | 12.773 | 205.1434 | 4.32 |  | 17.551 | 261.0449 | 4.14 |


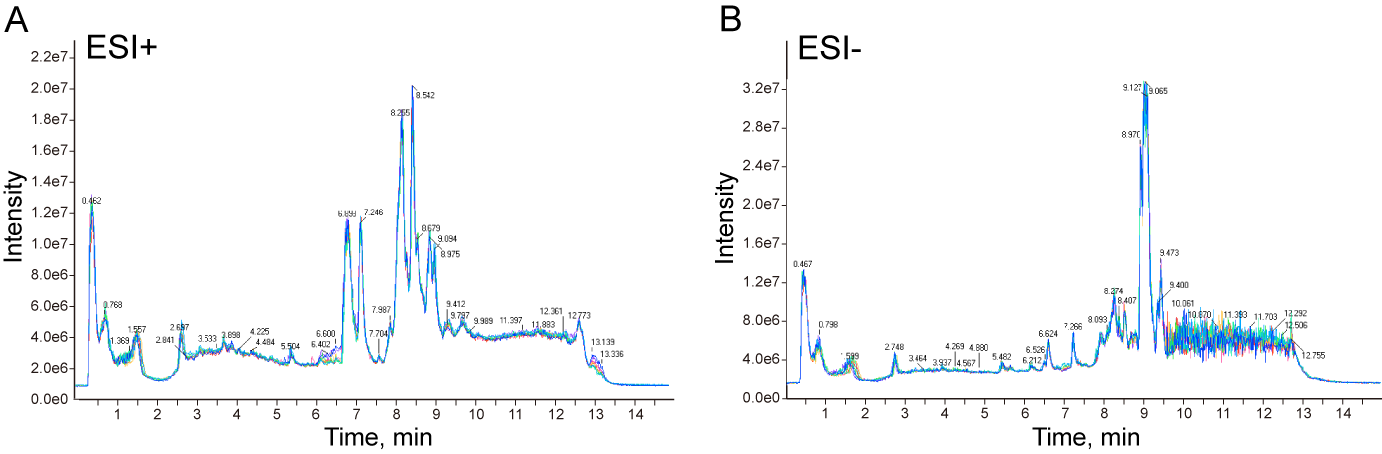


Fig. S1. TIC chromatograms of QC samples in our metabolomic analysis. (A) ESI+; (B) ESI-.


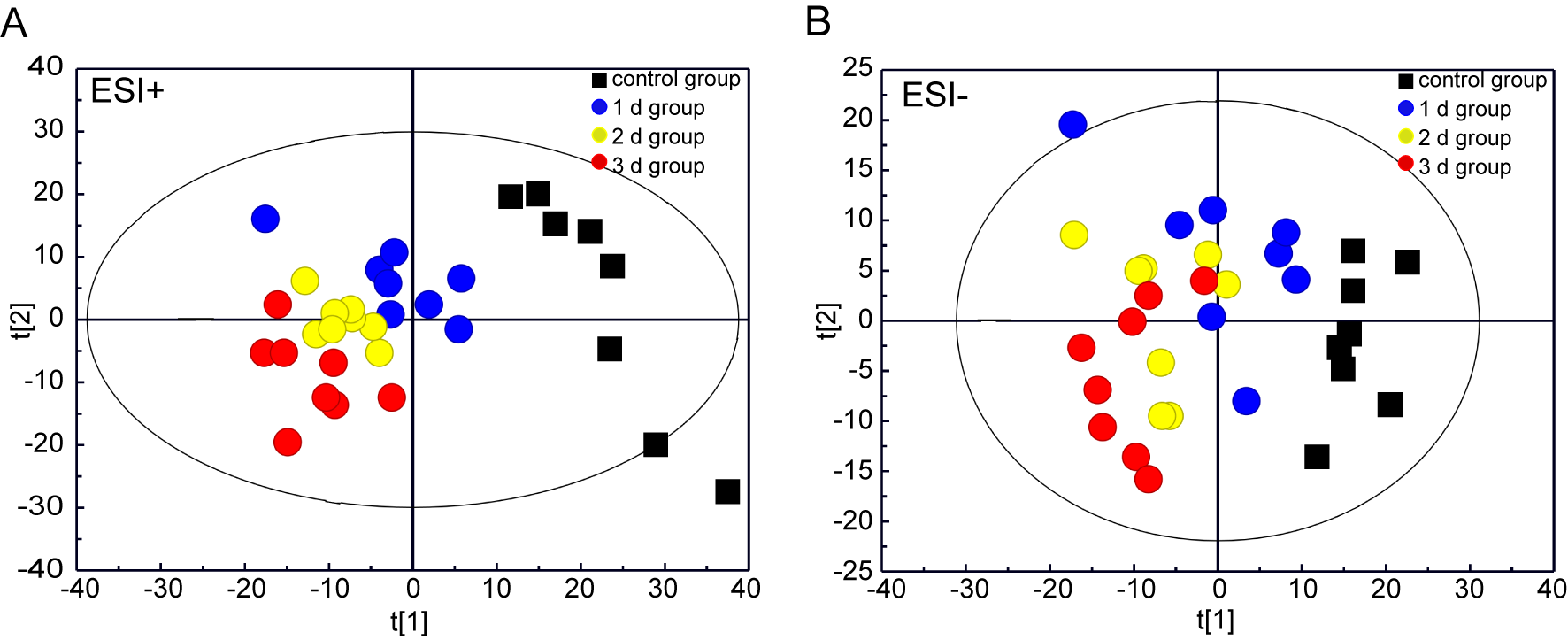


Fig. S2. Score plots for PCA analysis of UPLC/Q-TOF MS data from the 1 d, 2 d, 3 d and control groups. (A) ESI+; (B) ESI-.

、


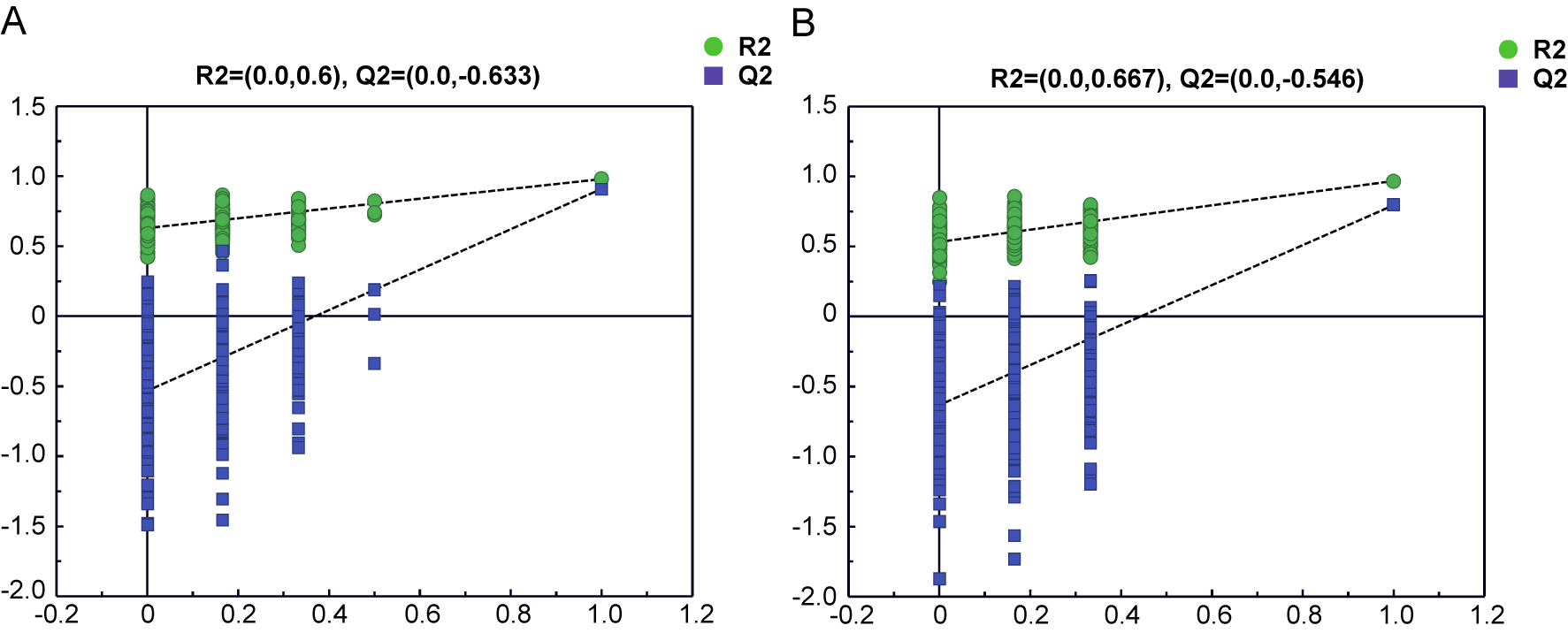


Fig. S3. Cross-validation plot of OPLS-DA mode of UPLC/Q-TOF MS data from the control and experimental groups with 300 times permutation tests.
